# Empirical methods for controlling false positives and estimating confidence in ChIP-Seq peaks

**DOI:** 10.1186/1471-2105-9-523

**Published:** 2008-12-05

**Authors:** David A Nix, Samir J Courdy, Kenneth M Boucher

**Affiliations:** 1 Huntsman Cancer Institute, Departments of Research Informatics, University of Utah, Salt Lake City, Utah, 84105, USA; 2 Oncological Sciences, University of Utah, Salt Lake City, Utah, 84105, USA

## Abstract

**Background:**

High throughput signature sequencing holds many promises, one of which is the ready identification of *in vivo *transcription factor binding sites, histone modifications, changes in chromatin structure and patterns of DNA methylation across entire genomes. In these experiments, chromatin immunoprecipitation is used to enrich for particular DNA sequences of interest and signature sequencing is used to map the regions to the genome (ChIP-Seq). Elucidation of these sites of DNA-protein binding/modification are proving instrumental in reconstructing networks of gene regulation and chromatin remodelling that direct development, response to cellular perturbation, and neoplastic transformation.

**Results:**

Here we present a package of algorithms and software that makes use of control input data to reduce false positives and estimate confidence in ChIP-Seq peaks. Several different methods were compared using two simulated spike-in datasets. Use of control input data and a normalized difference score were found to more than double the recovery of ChIP-Seq peaks at a 5% false discovery rate (FDR). Moreover, both a binomial p-value/q-value and an empirical FDR were found to predict the true FDR within 2–3 fold and are more reliable estimators of confidence than a global Poisson p-value. These methods were then used to reanalyze Johnson et al.'s neuron-restrictive silencer factor (NRSF) ChIP-Seq data without relying on extensive qPCR validated NRSF sites and the presence of NRSF binding motifs for setting thresholds.

**Conclusion:**

The methods developed and tested here show considerable promise for reducing false positives and estimating confidence in ChIP-Seq data without any prior knowledge of the chIP target. They are part of a larger open source package freely available from http://useq.sourceforge.net/.

## Background

Chromatin immunoprecipitation (chIP) is a well-characterized technique for enriching regions of DNA that are marked with a modification (e.g. methylation), display a particular structure (e.g. DNase hypersensitivity), or are bound by a protein (e.g. transcription factor, polymerase, modified histone), *in vivo*, across an entire genome [1]. Chromatin is typically prepared by fixing live cells with a DNA-protein cross-linker, lysing the cells, and randomly fragmenting the DNA. An antibody that selectively binds the target of interest is then used to immunoprecipitate the target and any associated nucleic acid. The cross-linker is then reversed and DNA fragments of approximately 200–500 bp in size are isolated. The final chIP DNA sample contains primarily background input DNA plus a small amount (<1%) of additional immunoprecipitated target DNA.

Several methods have been used to identify sequences enriched in chIP samples (e.g. SAGE, ChIP-PET, ChIP-chip [2-4]). One of the most recent utilizes high throughput signature sequencing to sequence the ends of a portion of the DNA fragments in the chIP sample. In a typical ChIP-Seq experiment, millions of short (e.g. 26 bp) sequences are read from the ends of the chIP DNA. The reads are mapped to a reference genome and enriched regions identified by looking for locations with a 'significant' accumulation of mapped reads. Calculating significance would be rather straight forward if the distribution of mapped reads were random in the absence of chIP (e.g. sequencing of input DNA). This does not appear to be true. The method of DNA fragmentation, preferential amplification in PCR, lack of independence in observations, the degree of repetitiveness, and error in the sequencing and alignment process are just a few of the known sources of systematic bias that confound naive expectation estimates.

Several methods have been developed to identify and estimate confidence in ChIP-Seq peaks. Johnson et al. used an ad hoc masking method based on their control input data and prior qPCR validated regions to set a threshold and assign confidence in their NRSF binding peaks [5]. Robertson et al. estimated global Poisson p-values for windowed data using a rate set to 90% the bp size of the genome. To estimate FDRs, a background model of binding peaks was generated by randomizing their STAT1 data and choosing a threshold that produced a 0.1% FDR [6]. Mikkelsen et al. took a remapping strategy that involved aligning every 27 mer in the mouse genome back onto itself to define unique and repetitive regions. For each ChIP-Seq dataset, "nominal" p-values were calculated by randomly assigning each read to a "unique region" and comparing the observed randomized 1 kb window sums to the real 1 kb window sums [7]. Mikkelsen et al. also employed a Hidden Markov Model that awaits description. Fejes et al. mention a Monte Carlo based FDR estimation based on read location randomization in their Find Peaks application note [8]. Lastly, Valouev et al. use a variety of promising enhancements (e.g. weighted windows/kernel density and read orientation) to call binding peaks from ChIP-Seq data and estimate FDRs base on control input [9]. Only the Johnson et al. method makes use of input data to control for localized systematic bias. This is unfortunate given the presence of clear systematic bias in ChIP-Seq data, see below. Additionally, none of the methods reported evaluation of their confidence estimations using spike-in data or simulated spike-in data where actual FDRs can be compared to estimated confidence metrics. This is critical for evaluating the usefulness of any novel ChIP-Seq peak discovery method.

## Results and discussion

In this paper we have 1) developed several methods to identify ChIP-Seq binding peaks while controlling for systematic bias 2) examined three methods for estimating statistical confidence in the peaks without prior knowledge 3) characterized these methods using both simulated spike-in data and a reanalysis of a published ChIP-Seq dataset and lastly, 4) created an open source software framework to support the development of next generation sequencing data analysis applications (see http://useq.sourceforge.net/). Included in the current USeq package are the low level ChIP-Seq analysis applications described here for converting mapped reads into chromosome specific summary tracks and enriched regions as well as numerous high level analysis applications for intersecting genomic regions, finding neighbouring genes, scoring binding sites, etc. A user guide, table of available applications, and other supporting documentation are available on the project website and with this manuscript, [see Additional file 1].

### Systematic bias

**Figure 1:**
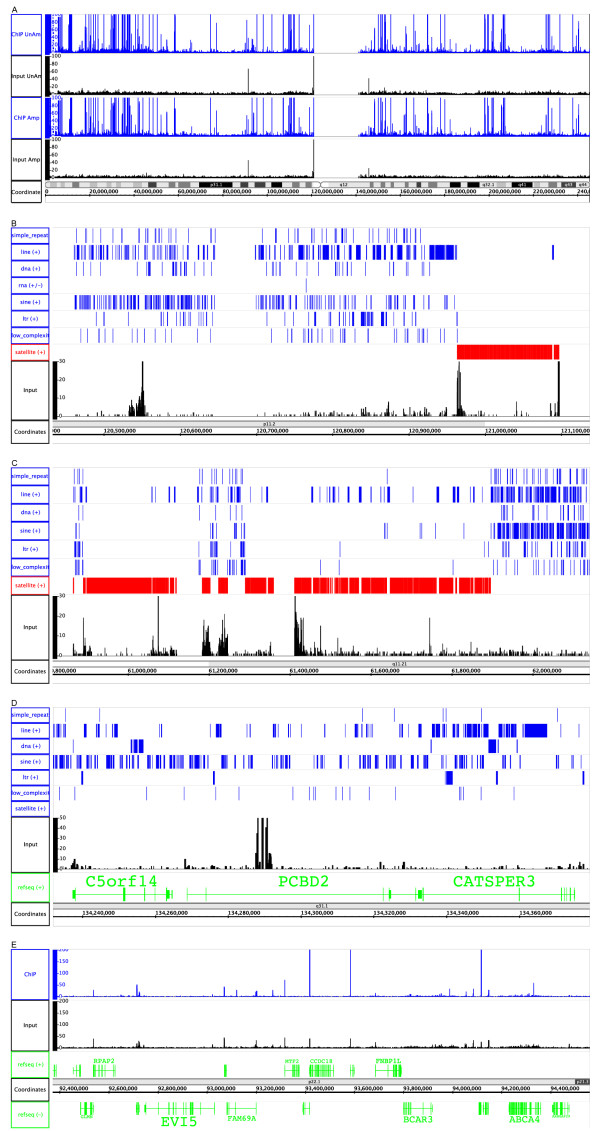
**Systematic bias**. Integrated Genome Browser display of chromosome 1 showing four window read count tracks derived from Johnson et al.'s NRSF *H. sapiens *ChIP-Seq data (A). The datasets were sub sampled to contain matching number of reads and the number of reads falling within a sliding 100 bp window plotted across each chromosome. Both the unamplified and amplified control input datasets show both obvious and subtle regions with an above random number of mapped reads. Expanded views of the input data track pericentric heterochromatic regions on chromosomes 1 (B) and 7 (C) along with UCSC's RepeatMasker tracks show that satellite (red) repeats overlap some but not all regions of apparent mapped sequence enrichment. This systematic bias is also apparent within genes and at transcription start sites (D). The degree of bias varies by dataset. For example, figure E, derived from Valouev et al.'s GABP ChIP-Seq data, shows very pronounced transcription start site read enrichment in the control input and chIP sample.

**Figure 2:**
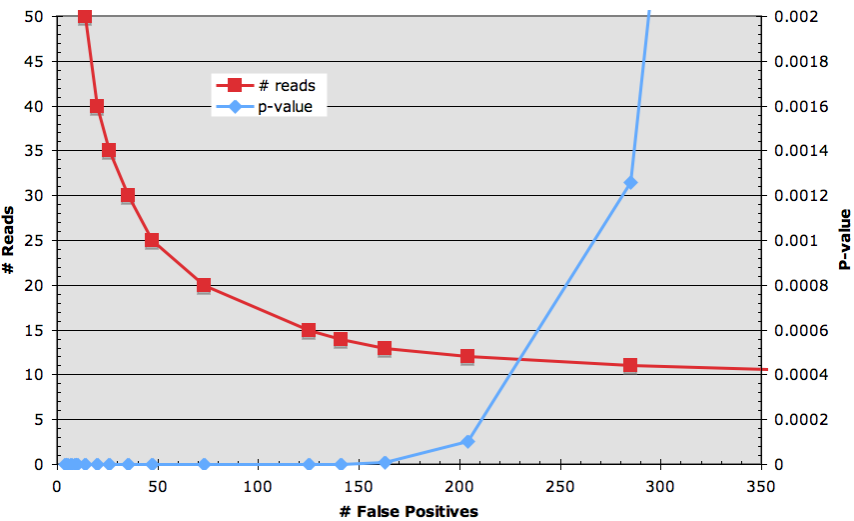
**Impact of systematic bias on the number of false positives**. The number of false positives due to systematic bias can be quite substantial. Figure 2 plots the number of false positives in the control unamplified input data from Johnson et al.'s NRSF study as a function of the number of window reads and Bonferroni corrected global Poisson p-values.

A visual inspection of several ChIP-Seq *control input *datasets [5,7,9] revealed clear evidence of non-random mapped read enrichment. The bias is in some cases obvious (e.g. spikes contained within most satellite repetitive regions adjacent to centromeric heterochromatin, figure 1A–C) and worth removing prior to analysis. The bias is also subtle (e.g. peaks within genes, figure 1D, at the transcription start sites of some genes, figure 1E, and peaks with no known associated annotation, figure 1B) and not so easily minimized. These false positives are seen in control input data and chIP data in both unamplified and PCR amplified datasets, figure 1A. If uncontrolled, the impact of these false positives can be quite substantial. Figure 2 shows the number of false positives in the Johnson et al. control unamplified input data as a function of the number of window reads and Bonferroni corrected global Poisson p-values. At a conservative p-value threshold of 0.001 (>10 reads per 350 bp window, by random chance < 1 read is expected per window), more than 250 false positives are obtained, at a threshold of 2 × 10^-14 ^(>20 reads), > 70 false positives are apparent.

### ChIP-Seq peak detection methods

The need to control for systematic bias motivated us to develop and test several methods to minimize the number of false positives using spike-in datasets. Spike-in datasets have proven to be instrumental in evaluating novel ChIP-chip[10] and expression microarray[11] analysis methods. They provide a known truth. As such, one can ask how many spike-ins and non-spike-ins (false positives) are recovered using a particular method at a given threshold. By fixing an acceptable FDR (# non-spike-ins/total # recovered regions) the method that returns more spike-ins is, by definition, better. Spike-in data is also useful for measuring the accuracy and consistency of novel confidence estimators. Presently, experimentally derived ChIP-Seq spike-in datasets do not exist. Therefore, we generated a close approximation by adding simulated ChIP-Seq reads to experimentally derived input control data. Our test datasets included two spike-in simulations that were created by adding 900 spike-in regions containing 2–60 reads each to human input data from Johnson et al. and 870 spike-in regions containing 1–87 reads to a combination of mouse input data and data showing little to no enrichment from Mikkelsen et al. The two datasets were made deliberately different to test the robustness of our methods against different data density, different alignment methods, and different levels of noise. The human dataset (low) represents one with low coverage; 1.6 million reads in each of the three samples: input 1, input 2, and input 3 + simulated chIP reads. The mouse dataset (high) represents one with relatively high coverage, 16 million reads in each sample, and likely more noise due to the inclusion of some low-level chIP enrichment.

**Figure 3:**
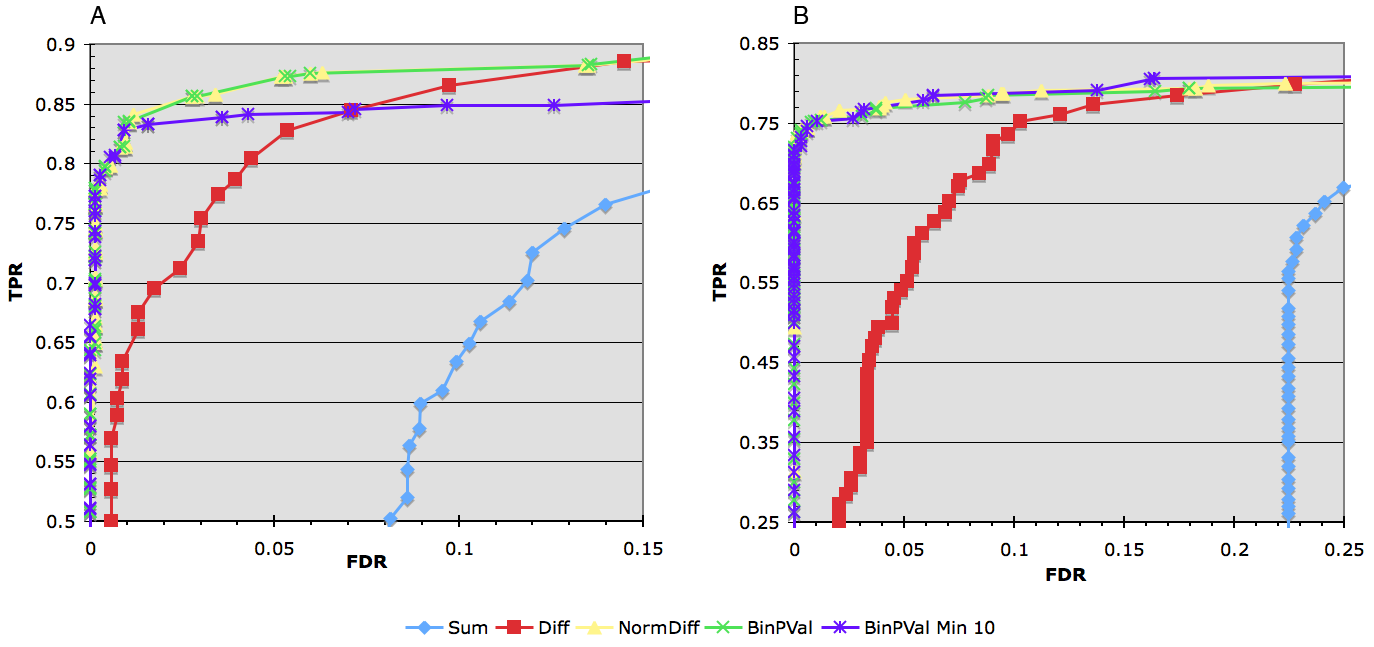
**Performance of different window summary statistics**. A comparison of the TPR against the FDR for four different window summary statistics associated with the low (A) and high (B) spike-in datasets. Each of the window scanning statistics (Sum: sum of the chIP reads within the window, no input; Diff: difference between the number of chIP reads and input reads; NormDiff: normalized difference, the difference divided by the square root of the sum; BinPVal: binomial p-value; BinPVal Min 10: binomial p-value using a prefiltered dataset where only windows with 10 or more total reads from the chIP and input datasets were used to score the mapped spike-in data. Multiple sets of enriched regions where generated over a range of thresholds. Each set was then intersected with the appropriate spike-in key and the TPR and FDR plotted.

Four different peak identification methods were compared using the two simulated ChIP-Seq datasets. Each method made use of a sliding window (350 bp) to generate summary scores for all interrogated regions in the genome. Overlapping windows were combined into candidate binding peaks by merging those that exceed a given threshold. Binding peaks were then scored for intersection with the spike-in key and the true positive rate (TPR) and the FDR calculated. Figure 3 shows a plot of TPRs against FDRs over a variety of thresholds for each of the four methods. The "sum" method uses no input control data but simply sums the number of reads falling within each window. The "difference" method is a subtraction of the sum of the reads in the chIP data minus the sum of the reads in the input control data for each window. The "normalized difference" method takes the difference and divides it by the square root of the sum, an estimation of the standard deviation. Lastly, binomial p-values were calculated as described in the methods. Of the four, the normalized difference and binomial p-value out performs the others at all FDRs with the normalized difference slightly better in the small dataset. In situations where control data is available, regions in the genome with significant global Poisson p-values can be identified and removed prior to windowing. Application of this pre filter significantly improved the difference window score making it essentially equivalent to the normalized difference score and slightly improved the performance of the other window tests (data not shown). However, the risk of picking an inappropriate p-value threshold for pre filtering the data may preclude its usefulness.

### ChIP-Seq peak confidence estimations

**Figure 4:**
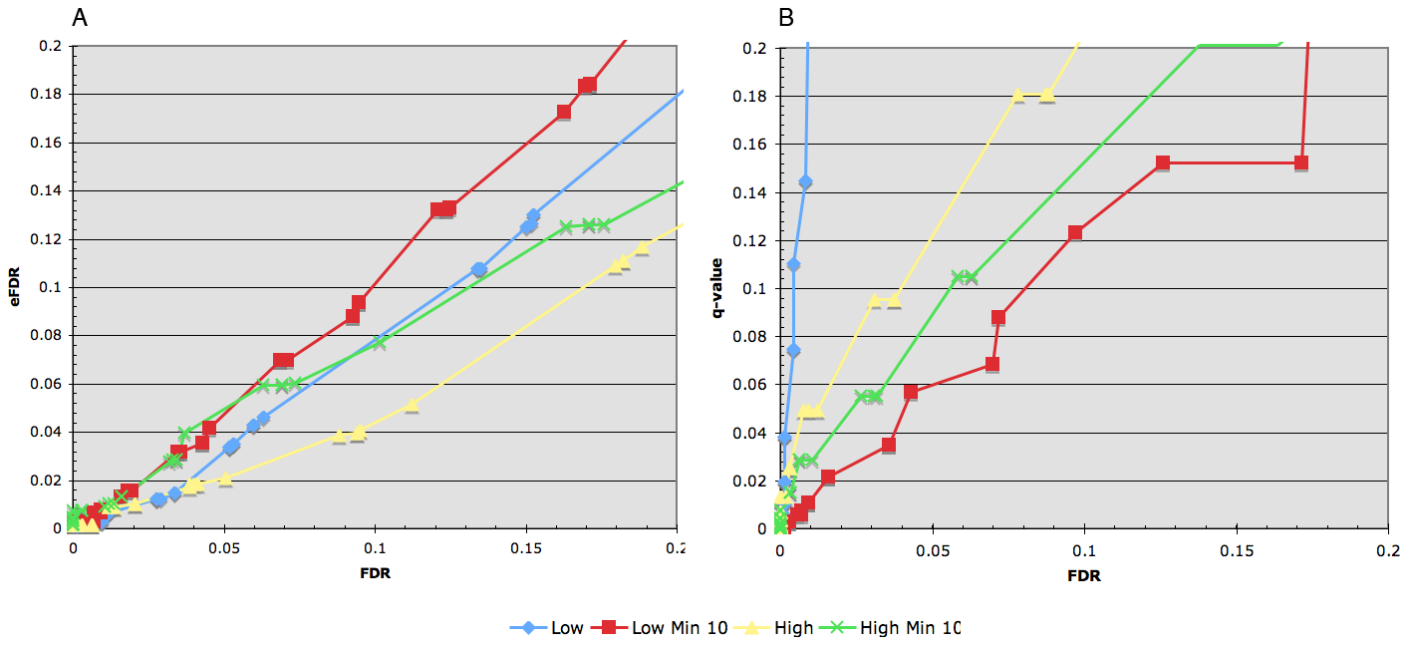
**Performance of two confidence estimations**. A comparison of two FDR estimations against the real FDR for the two spike-in datasets with low and high number of reads. Empirical FDRs (eFDR) were calculated and plotted (A) for a variety of thresholds by dividing the number of control enriched regions (input1 vs. input2) by the number of chIP enriched regions (chIP vs. input2). The actual FDR was calculated by intersecting each enriched region set with the spike-in key. The q-value FDR estimation (B) is made by calculating binomial p-values for each window and applying the Storey q-value FDR approximation. For each of the spike-in datasets, windows were generated using two different minimum number of reads, either 1 or 10. The latter represents a filtered dataset that improves the q-value estimation at the cost of test sensitivity (see also Figure 2A).

A second goal in our study was to develop and evaluate methods for estimating confidence in ranked lists of putative binding peaks. Binding peaks passing a set threshold will contain both real and false positives. Estimating the degree of false positives in a given list, an FDR, is a critical step in generating useful ChIP-Seq data. We evaluated two methods for their accuracy and reliability using the two simulated spike-in datasets (Figure 4). The first makes use of normalized difference score window data to generate sets of enriched regions. Empirical FDRs (eFDRs) are calculated at each test normalized difference score by dividing the number of control enriched regions (input 1 vs. input 2) by the number of test enriched regions (chIP vs. input 2). Figure 4a compares the eFDRs against the actual FDRs for the two spike-in simulation datasets. In both cases, the eFDR underestimates the actual FDR by < ~2 fold (e.g. an FDR of 0.01 = eFDRs of 0.005 – 0.009, an FDR of 0.05 = eFDRs of 0.02 – 0.05, an FDR of 0.1 = eFDRs of 0.045 – 0.01).

The second FDR approximation (Figure 4b) uses Storey's [12] q-value method to convert window binomial p-values to q-values. At the core of the q-value conversion is the assumption that under the null, input p-values are uniformly distributed. This presents a problem with ChIP-Seq data. For small datasets, a significant number of regions contain < 5 reads, their binomial p-values are not continuous but produce discrete values (e.g. 0.25, 0.5, 1), spikes in the p-value distribution, and a poor q-value FDR estimation, see figure 4b. To more closely approximate a uniform distribution, windows with < 10 reads are removed prior to converting p-values. This significantly improves the q-value FDR estimation but does affect the test sensitivity with small datasets, see figure 3a. With both datasets, binomial p-values converted to q-values overestimated the actual FDR by < ~3 fold (e.g. an FDR 0.01 = q-value 0.01 – 0.03, an FDR 0.05 = q-value 0.06 – 0.09, and an FDR of 0.1 = q-value 0.12–0.15).

Each of the two confidence estimators has its own advantages and disadvantages. The eFDR underestimates the actual FDR and requires twice the number of control input reads to generate a null distribution. Yet the eFDR has no set minimum data size and can thus be used with low read density datasets. The q-value FDR overestimates the true FDR, requires a minimum of 10 reads in each window, and half the input control data. It is most useful for high read density data with matching input control data. In practice, we use both FDR estimations to get an approximate range of likely confidence for a given list of enriched regions.

Lastly, the viability of using global Poisson p-values was assessed with the simulated spike-in data sets. This method assumes random read distribution under the null hypothesis. It does not make use of input control data to down weight localized systematic bias. As a result, it performed rather poorly. Real FDRs of < 0.08 could not be achieved at any threshold due to the presence of high numbers of false positives. It is useful in situations where control data is unavailable but otherwise, it should be avoided.

### Analysis of the Neuron-Restrictive Silencer Factor ChIP-Seq data

Using the methods developed here, we reanalyzed Johnson et al.'s NRSF ChIP-Seq data. Currently, this is the only published ChIP-Seq dataset with control input data and extensive qPCR validated regions. The authors did not perform an input subtraction but used the control data to exclude regions with high numbers of control reads (> = 20%), a hard mask. To set a threshold, they constructed ROC curves using 83 known qPCR verified NRSF binding regions and 130 qPCR negative regions and chose a threshold (> = 13 chIP reads) that gave high sensitivity and specificity. Their PCR amplified and non-amplified datasets were processed independently and those regions common to both were selected to represent likely NRSF binding regions.

To reanalyze the data, we combined the two datasets, ran them through ScanSeqs with a window size of 300 bp and selected a normalized difference score threshold that produced a ranked list of 1944 regions that were subsequently trimmed to match the number of non redundant regions found in Johnson et al.'s supplementary material, 1932. Intersections were then made between the enriched region lists to evaluate ScanSeqs performance. The two lists were quite similar (93%) and intersected the same number of NRSF qPCR positives (87%) and negatives (4%). The authors state that their list is likely a conservative estimate of the true number of NRSF binding regions. The eFDR and q-value FDR associated with the normalized score threshold used in generating the 1932 regions, 3.5 × 10^-4 ^and 1.4 × 10^-6^, agree with this estimate. To get an idea of the number of regions with an FDR of less than 0.01 we set thresholds and generated enriched regions using an eFDR of 0.005 or a q-value of 0.01 to yield 3223 and 4731 regions respectively. These data and a reanalysis of Mikkelsen et al.'s [7] and Barski et al's [13] histone modification, RNA polymerase II, and CTCF data are posted on our DAS/2 server http://bioserver.hci.utah.edu:8080/DAS2/das2 and best accessed using the Integrated Genome Browser (IGB, http://bioserver.hci.utah.edu/BioInfo/index.php/Software:IGB).

### Implementation

The USeq package contains more than 25 command line applications written in Java for portability, speed, and collaborative development. It makes use of R http://www.r-project.org/ and Storey's q-value package http://genomics.princeton.edu/storeylab/qvalue/. In addition to generating standard text based data summary and result file types (e.g. gff, xls), extensive use of the Affymetrix binary bar file format has been made for direct viewing in IGB and optimized distribution using the DAS/2 protocol and the GenoViz DAS2 server, see http://bioserver.hci.utah.edu/BioInfo/index.php/Software:IGB and http://genoviz.sourceforge.net/. USeq is distributed under an open source BSD license. Documentation related to analysis usage, available applications, command line menus, and output file type descriptions are included [see Additional file 1] and on the USeq project web site http://useq.sourceforge.net/.

### Testing

USeq has been tested primarily on Red Hat Enterprise Linux 5 and Mac OS X with limited evaluation on Windows XP. A typical analysis run takes < 2 hrs for 40 million mapped chIP and control reads on a dual processor 64 bit HP server with 8 Gb RAM. The majority of time is spent writing out the binary bar graph files. Run time without bar file write out is < 20 min.

## Conclusion

The ability to control for systematic bias and accurately estimate confidence in chIP peaks are two critical steps in generating useful data from chIP experiments. Here we have developed novel methods to reduced systematic bias by directly comparing mapped enrichment at each genomic loci in the chIP data against control input data. This allows proportional scoring of genomic regions without imposing a hard mask. More importantly, we developed and characterized two methods for estimating FDRs associated with chIP enrichment that do not rely on extensive qPCR validation. Much is unknown about this new ChIP-Seq data type. Empirical methods, such as detailed here, are proving to be a good first approximation. As more becomes known, modelling of the ChIP-Seq detection and mapping process may replace the need for input controls. Until that point, we strongly recommend the generation of input datasets to control for systematic bias and enable ChIP-Seq peak confidence estimation. These input control datasets are likely reusable barring major changes in the chIP sample preparation and alignment methods.

## Methods

### Bonferroni corrected global Poisson p-values

To estimate confidence in ChIP-Seq data without using an input control, a p-value can be calculated using the Poisson formula.

$p_{i}=1-\sum_{j=0}^{Yi-1}\frac{\lambda e^{-\lambda }}{j!}$ where **Y_i _**is equal to the number of reads falling within the window **i **and **λ **equal to the expected number of reads to map to the window by random chance (size of the window * total number reads/effective genome size (0.9 * genome size)), see also Robertson et al. A conservative multiple testing correction is made following Bonferroni [14] by multiplying each p-value by the number of window tests.

### Spike-in Data Set Generation

An application was developed to simulate single binding site chIP regions. It works by randomly selecting center positions from a genome. These are expanded to a maximum defined size (500 bp) and then filtered to remove regions with a RepeatMasker base content of greater than 0.2 and a fraction of non GATC bases greater than 0.5. For each remaining region, random fragments are generated about each center position from 150 to 500 bp in size. From each simulated fragment, each end is taken as a read and each base in the read mutated according to the published per cycle error frequency[15]. Reads are then aligned to the genome.

For the human spike-in dataset, 1000 regions with 1000 simulated chIP fragments were selected producing 2000 reads each. These were mapped to hg17 using the ELAND Extended aligner from Illumina. Only those regions with greater than 1000 mapped reads were chosen for use in generating the spike-in set 60 groups containing 30 spike-in regions were created. For each of the groups, from 2 to 60 reads were randomly drawn from each of the 30 residing spike-in regions. These represent 900 spike-in regions containing 2 to 60 reads, 27,900 total. To create the actual spike-in datasets, Johnson et al.'s control input data was combine, randomized and split in thirds, 1,698,713 reads each. To one of the thirds, the reads from the 900 spike-in regions were added. This represents the simulated chIP data, the other two, input data sets.

In a similar fashion, the mouse spike-in dataset was generated. Reads from 1000 regions with 1000 fragments were mapped to mm8. Regions with greater than 500 mapped reads were used to derive 87 groups, each with 10 regions, 870 total containing 1 to 87 randomly drawn reads, 38,280 total. To generate a larger simulated input dataset, data from Mikkelsen et al. that showed little to no significant enrichment (ES.H3, ES.K9, ES.RPol, ESHyb.K9, MEF.K9, NP.K9, NP.K27, and NP.K36) were pooled along with their actual whole cell extract input data (ES.WCE, MEF.WCE, and NP.WCE), randomized, and split in thirds, 16,383,950 reads each. For download of the spike-in datasets, [see the website http://bioserver.hci.utah.edu/SupplementalPaperInfo/2008/Nix_EmpiricalMethods/SpikeInData.zip for additional file 2].

### Window Scoring Datasets

The following procedure was followed for processing mapped ChIP-Seq data.

1) Mapped reads were sorted by chromosome and strand and their centered positions saved to disk using the binary bar file format. When sequencing the termini of chIP fragments, a bimodal distribution of mapped reads is created that needs to be centered to approximate the true IP site by shifting the read positions 3' by 1/2 the mean fragment length. For simulated spike-in datasets, the mean fragment length is known and read positions were shifted, 175 bp. For real ChIP-Seq datasets, Solexa sequencing displays a significant bias toward sequencing shorter fragments (data not shown). As such, the number of bases needed to center the chIP data needs to be determined using stranded datasets (60 bp for the Johnson et al. NRSF dataset, 110 bp for the Mikkelsen datasets).

2) The first step ScanSeqs takes to compare two mapped datasets is to trim one of the datasets to match the other by randomly removing reads. For calculating an empirical FDR, the number of reads in the input dataset needs to be twice the number of reads in the chIP data. For other comparisons, the number of reads needs to be made equal between the datasets.

3) To score regions for accumulation of mapped reads, overlapping windows with a maximum size are created as follows. All the data, treatment and control, are merged. The first mapped read is identified and the last read within a defined maximum window size (e.g. 350 bp) is used to define the start and stop of the window. The window is then advanced to the next mapped read and the last read falling within the maximum window size examined. If its position is the same as the prior window termini, the window is skipped since this collection of reads is a subset of the prior. The start and stop positions for "unique" window on each chromosome are then used to calculate several window level statistics from the chIP and input datasets.

*a. Sum of chIP read scores and the sum of input read scores: *In this first implementation of ScanSeqs, each read score is set to 1. The sum is thus the number of reads.

*b. Window level binomial p-*values: Let **Y **be the number of test reads and **X **the number of input reads within a particular region and **S **= **X **+ **Y**.

Given **S**, the **Y **data is assumed to have a binomial distribution with a probability parameter of 0.5 and number of observations **S**. A conditional one-sided p-value can be calculated using the R function, **P **= pbinom (**Y**-1, **S**, 0.5, lower.tail = FALSE).

*c. A normalized window score: *when **N = M**, a normalized score is given by **S_i _**= **(Y_i_- X_i_)/(Y_i_+ X_i_)^1/2 ^**where the denominator is an estimation of the standard deviation.

*d. eFDR: *Empirical FDRs are calculated for each normalized window score by generating enriched regions, see below, from two datasets, the test dataset (chIP vs. input2) and the control dataset (input1 vs. input2). The **eFDR **at **S_i _**= **(# control enriched regions)/(# test enriched regions)**. At extreme thresholds, the eFDR can actually increase with increasing stringency. In these cases, the previous, lower stringency eFDR is assigned.

4) To create lists of enriched regions/candidate binding peaks, a threshold and a maximum gap are chosen. Windows passing the threshold and with ends within the maximum gap are merged. Scores from the best window are assigned to the enriched region and used to create a ranked list of binding peaks.

### Comparison of Spike-in Simulated ChIP Datasets with the Key

To evaluate the performance of different window summary statistics and confidence estimators, rank lists of candidate binding peaks were created at multiple thresholds and intersected with the known list of spike-in regions. An intersection was scored if the known binding region abutted or overlapped the predicted binding region. For each list, the TPR and FDR were calculated. TPR is simply the number of intersected spike-in regions divided by the total. The FDR is the total number of predicted binding regions minus the number of intersected spike-in regions divided by the total number of predicted binding regions. As with the eFDRs, lists made with increasing stringent thresholds occasionally show an increased FDR. In these cases, the previous FDR is assigned to the list.

## Authors' contributions

DN developed the methods, the spike-ins, the USeq software package, and drafted the manuscript. SC participated in the design and coordination of the study and reviewed the manuscript. KB provided key statistical insight and pilot implementation of the binomial p-value and normalized difference scoring metrics as well as in the design of the study and review of the paper. All authors have read and approved the final manuscript.

## Supplementary Material

Additional File 1 **A variety of html documents from the USeq web site detailing the available applications, their best usage, output file type descriptions, command line menus, etc**. Click here for file
