# Supplementary material for: Empirical methods for controlling false positives and estimating confidence in ChIP-Seq peaks
Source: BMC Bioinformatics. 2008 Dec 5;9:523. doi: 10.1186/1471-2105-9-523 (PMC2628906; doi:10.1186/1471-2105-9-523)
Supplement: Additional File 1 — A variety of html documents from the USeq web site detailing the available applications, their best usage, output file type descriptions, command line menus, etc. [file 1471-2105-9-523-S1.zip › USeqUserGuides/applications.html]

USeq Applications


# Core Applications

Launch these applications using the -jar option, for example, 'java -jar pathTo/USeq/Apps/FileSplitter' .  
Click the name to see the command line menu.

|  |  |
| --- | --- |
| Sequencing Analysis Apps | |
| Eland Parser Splits and converts stand alone or Solexa pipeline Eland Extended xxx\_export.txt and xxx\_sorted.txt alignment files into center position alignment scored binary PointData xxx.bar files. | |
| Eland Multi Parser Parses an Eland xxx.eland\_multi.txt file tabulating hits to each fasta entry. Good for scoring hits to a transcriptome where every fasta entry represents a different gene. | |
| Eland Sequencing Parser Generates sequencing summary tracks for each base and a called consensus from Eland export and sorted alignment files. | |
| Tag 2 Point Splits and converts tab delimited text (chr start stop ... strand, e.g. xxx.bed) text files into center position binary xxx.bar PointData files used by ScanSeqs and IGB for analysis and visualization respectively. Very fast loading. Small size. | |
| QC Seqs QCSeqs takes directories of chromosome specific PointData xxx.bar.zip files that represent replicas of signature sequencing data, merges the strands, uses a sliding window to sum the hits, and calculate Pearson correlation coefficients for the window sums between each pair of replicas. | |
| Filter Point Data FPD drops observations from PointData that intersect a list of regions (e.g. repeats). | |
| Sub Sample Point Data Creates a random sub sampling of Point Data. Useful for matching treatment and control datasets. Note, this is automatically performed in ScanSeqs. | |
| Scan Seqs Takes chromosome specific PointData xxx.bar.zip files and uses a sliding window to calculate smoothed window scores. These are saved as point and heat map/ stairstep xxx.bar.zip graph files. If no control data is provided, Bonferroni corrected p-values are estimated using a global Poisson distribution. Otherwise, binomial p-values and q-value FDRs are estimated based on the treatment vs control window scores. Lastly, empirical FDRs can be estimated by generating a null distribution of control - control windows and scoring each treatment - control window relative to the null. In this case, one should provide >2x the # of control reads relative to the treatment to prevent sub sampling of the treatment observations. | |
| Defined Region Scan Seqs Similar to Scan Seqs. Takes chromosome specific PointData xxx.bar.zip files and extracts scores under each region and calculates several statistics including a binomial p-value and Storey corrected q-value. If a gene table is provided, scores under each exon are summed to give a whole gene summary. Control data is optional but recommended. | |
| EnrichedRegionMaker Combines SmoothedWindowInfo xxx.swi files from ScanSeqs into larger Enriched Regions/ Binding Peaks given a score index, a minimum score, and a maximum gap. Can also be used to find the best peak within each Enriched Region. | |
|  | |
| General Analysis Apps | |
| Find Neighboring Genes FNG takes a list of genes in UCSC Gene Table format and intersects them with a list of regions finding the closest gene to each region as well as all of the genes that fall within a given neighborhood. | |
| Intersect Regions Performs an intersection analysis on lists of genomic regions, uses random regions matched for GC content, length, array interrogated regions, and chromosome to calculate an enrichment over random and p-value. Also generates a distance to nearest region distribution histogram. | |
| Intersect Lists Intersects two lists (of gene names) and using randomization, calculates the significance of the intersection and the fold enrichment over random. | |
| Ranked Set Analysis Performs an intersection analysis on lists of ranked regions creating a visual box-line-box representation as well as a rank based % intersection graph. | |
| Correlation Maps CM creates correlation maps from gene expression data to look for physical gene clusters (aka gene expression neighborhoods, chromosome territories). | |
| Score Chromosomes Scores a genome for hits to a transcription factor binding matrix, LLPSPM. | |
| Score Parsed Bars Given a list of regions and a directory of graph data in bar format, extracts all the values under each region, calculates their mean and compares it to a random background model to generate a p-value for the associated means. | |
| Score Sequences Scores a multi-FASTA file of sequences for hits to a transcription factor binding matrix, LLPSPM. | |
|  | |
| Utility Apps | |
| Bar 2 Gr Converts xxx.bar files to text xxx.gr files. | |
| Gr 2 Bar Converts chromosome specific xxx.gr.zip files (position score) to binary chromosome specific xxx.bar files. | |
| Sgr 2 Bar Converts xxx.sgr.zip files (chr position score) to binary chromosome specific xxx.bar files. | |
| Wig 2 Bar Converts variable step and fixed step xxx.wig(Var) files to chrom specific bar files. | |
| Primer 3 Wrapper Wrapper for the primer3 (http://frodo.wi.mit.edu/primer3/) application. Extracts sequence, formats for primer3, executes, and parses the output to a spreadsheet. Useful for bulk qPCR primer picking. Yes, you do need to validate your results. | |
| Export Intergenic Regions Takes a GFF file and exports regions not covered by any annotation, the intergenic regions. | |
| Export Intronic Regions Takes a UCSC gene table and exports the most conservative estimate of intronic sequence. | |
| Fetch Genomic Sequences Given a file containing genomic coordinates, fetches and saves the sequence. | |
| Convert Fasta 2 GC Boolean Converts fasta sequences to GC boolean arrays for use by other applications. | |
| Make Splice Junction Fasta MSJF creates a multi fasta file containing sequences representing all possible linear splice junctions. | |
| Make Transcriptome Creates a multi fasta file containing exonic sequence for every gene plus splice junctions. | |
| File Cross Filter FCF take a column in the matcher file and uses it to parse the rows from other files. Useful for pulling out and printing in order the rows that match the first file. | |
| File Match Joiner FMJ loads a file and a particular column containing unique entries, a key, and then appends the key line to lines in the parsed file that match a particular column. Usefull for appending chromosome coordinates to snp data based on a comon ID, etc. | |
| File Joiner Joins many text files together, paying attention to avoid fusing the last and first lines from two files. Use to combine the remapped TPMap file. | |
| File Splitter Splits a text file into many files given a number of lines, use to split the TPMap file for TPMapOligoBlastFiltering. | |
| Print Select Columns Spread sheet/ tab delimited file manipulations. | |
