# Supplementary material for: Empirical methods for controlling false positives and estimating confidence in ChIP-Seq peaks
Source: BMC Bioinformatics. 2008 Dec 5;9:523. doi: 10.1186/1471-2105-9-523 (PMC2628906; doi:10.1186/1471-2105-9-523)
Supplement: Additional File 1 — A variety of html documents from the USeq web site detailing the available applications, their best usage, output file type descriptions, command line menus, etc. [file 1471-2105-9-523-S1.zip › USeqUserGuides/cmdLnMenus.html]

USeq Command Line Menus 

# Command Line Menus

Bar2Gr  
CorrelationMaps  
ConvertFasta2GCBoolean  
DefinedRegionScanSeqs  
EnrichedRegionMaker  
ElandMultiParser  
ElandParser  
ElandSequenceParser  
ExportIntergenicRegions  
ExportIntronicRegions  
FetchGenomicSequences  
FindNeighboringGenes  
FileCrossFilter  
FileMatchJoiner  
FileJoiner  
FileSplitter  
FilterPointData  
Gr2Bar  
IntersectLists  
IntersectRegions  
MakeSpliceJunctionFasta  
MakeTranscriptome  
Primer3Wrapper  
PrintSelectColumns  
RankedSetAnalysis  
ScanSeqs  
ScoreChromosomes  
ScoreParsedBars  
ScoreSequences  
Sgr2Bar  
SubSamplePointData  
Tag2Point  
QCSeqs  
Wig2Bar  

```
**************************************************************************************
**                                 Bar2Gr: Nov 2006                                 **
**************************************************************************************
Converts xxx.bar to text xxx.gr files.

-f The full path directory/file name for your xxx.bar file(s).

Example: java -Xmx1500M -jar pathTo/T2/Apps/Bar2Gr -f /affy/BarFiles/ 

**************************************************************************************
```

```
**************************************************************************************
**                           Correlation Maps:    Nov 2007                         **
**************************************************************************************
CM calculates a correlation score for each window of genes and using permutation, an
empirical p-value.  The correlation score is the mean of all pair Spearman ranks for
the gene expression profiles in each window. If a single value is given (unlogged!) for
each gene, a mean of the scores within each window is calculated.

To calculate p-values, X randomized datasets are created by shuffling the expression
profiles between genes, windows are scored and pooled.  P-values for each real
score are calculated based on the area under the right side of the randomized score
distribution. In addition to a spread sheet report summary, heat map xxx.bar files
for the p-values and mean correlation are created for visualization in IGB.
Note, this analysis is not stranded.  If so desired parse lists appropriately.

Parameters:
-f The full path file name for a tab delimited gene file (name,chr,start,stop,scores)
-o Region filter file, full path file name for a tab delimited region file to use in
      removing genes from correlation analysis. (chrom, start, stop).
-g Genome version for IGB visualizations (e.g. C_elegans_May_2007).
-w Window size, default is 50000bp. Setting this too small may exclude some regions.
-n Minimum number of genes required in each window, defaults to 3. Setting this too
       high will exclude some regions.
-r Number random trials, defaults to 100

Example: java -Xmx256M -jar pathTo/T2/Apps/CorrelationMaps -f /Mango/geneFile.txt
       -w 30000 -n 2 -o /Mango/operons.txt

**************************************************************************************
```

```
**************************************************************************************
**                       Convert Fasta 2 GC Boolean: Aug 2008                       **
**************************************************************************************
Converts fasta file(s) into serialized boolean[]s where every base g or c is true all
others false. Will also work with xxx.binarySeq files.

Required Parameters:
-f Full path name for the xxx.fasta file or directory containing such.

Example: java -Xmx2000M -jar pathTo/Apps/ConvertFasta2GCBoolean -f /affy/Fastas/

**************************************************************************************
```

```
**************************************************************************************
**                           DefinedRegionScanSeqs:  June 2008                      **
**************************************************************************************
Takes chromosome specific PointData xxx.bar.zip files and extracts scores under each
region and calculates several statistics including a binomial p-value and Storey
corrected q-value.  If a gene table is provided, scores under each exon are summed to
give a whole gene summary. Control data is optional but recommended.

Options:
-s Save directory, full path.
-r RefFlat or RefSeq UCSC gene table file, full path. See,
       http://genome.ucsc.edu/cgi-bin/hgTables
-b (Or) a bed file (chr, start, stop,...), full path, of regions.
-t Treatment Point Data directories, full path, comma delimited. These should
       contain chromosome specific xxx.bar.zip files. If needed, strands will be
       merged. 
-c (Optional) Control Point Data directories, ditto. 
-q Provide a full path to R with installed q-value library. See
       http://genomics.princeton.edu/storeylab/qvalue/
-l Enter # of mappable bases, typically 0.9 * bp size genome for complex organisms.
       Human 3253037807 * 0.9 = 2927734026
       S.cerevisiae 12162996 * 0.99 = 12041366
       See 'All genomes' http://www.ensembl.org

Example: java -Xmx1500M -jar DefinedRegionScanSeqs -t /Data/PolIIRep1/,/Data/PolIIRep2/
      -c /Data/Input1/,Data/Input2/ -s /Data/PolIIResults -r /Anno/refFlatHg17.txt 
      -l 2927734026 -e -q /usr/bin/R 

**************************************************************************************
```

```
**************************************************************************************
**                       Enriched Region Maker: September 2008                      **
**************************************************************************************
ERM combines SmoothedWindowInfo xxx.swi files into larger EnrichedRegions given a
score index, a minimum score, and a maximum gap. If treatment/control PointData are
provided, the best peak within each enriched regions will be identified.

Options:

-f Full path file name for the serialized SmoothedWindowInfo[] xxx.swi array, if a
      directory is specified, all xxx.swi files will be processed.
-s Minimal score, one or more, comma delimited, no spaces.
-i Score index to use in thresholding windows. Leave blank to print indexes.
-g Maximum allowable bp gap between windows, defaults to 300.
-n Make given number of ERs, one or more, comma delimited, no spaces.
-e Save serialized ER[] arrays.
-r Enter a full path tab delimited regions file name (chr start stop) to use in
      removing intersecting windows. Coordinates are assumed to be zero based and end
      inclusive.
-t Treatment Point Data directories, full path, comma delimited. These should
       contain chromosome specific xxx.bar.zip files. 
-c Control Point Data directories, ditto. 
-w Window size in bps for best peak identification, defaults to 50.

Example: java -Xmx500M -jar pathTo/USeq/Apps/EnrichedRegionMaker -f /solexa/zeste.swi
      -s 30,60 -i 1 -g 50 

**************************************************************************************
```

```
**************************************************************************************
**                          Eland Multi Parser: October 2008                        **
**************************************************************************************
Parses an Eland xxx.eland_multi.txt alignment file tabulating hits to each fasta entry.
Good for scoring hits to a transcriptome where every fasta entry represents a
different gene.

-f The full path directory/file name of your xxx.eland_multi.txt(.zip) file(s). Files
      will be merged.
-r Full path file name for saving the results.


Example: java -Xmx1500M -jar pathToUSeq/Apps/ElandMultipParser -f 
      /data/MultiFiles/ -r /data/transcriptomeResults.xls 

**************************************************************************************
```

```
**************************************************************************************
**                              ElandParser: June 2008                              **
**************************************************************************************
Splits and converts Eland Extended xxx_export.txt(.zip) or xxx_sorted.txt(.zip) files
into center position alignment scored binary xxx.bar files. Coordinates are in
interbase coordiantes (zero based, end excluded). These can be directly viewed in IGB.

-v Versioned Genome (ie hg18, dm2, ce2, mm8), see UCSC Browser,
      http://genome.ucsc.edu/FAQ/FAQreleases.
-m Minimum aligment score, Phred scale, defaults to 13. Not used with stand alone.
-f The full path directory/file name of your xxx_export.txt(.zip) or
      xxx_sorted.txt(.zip) file(s).
-r Full path directory name for saving the results, defaults to export.txt parent.
-s Shift centered position N bps 3' to accomodate chIP-seq fragment size. Stranded.
      Note, this is far less than 1/2 the expected fragment size, determine best
      value by visual inspection of likely positives. Defaults to 0. If you plan on
      filtering your PointData, don't shift their positions, do it in the filter app.
-p Parse stand alone Eland output file.

Example: java -Xmx1500M -jar pathToUSeq/Apps/ElandParser -f /Solexa/Run7/
     -v hg18 -s 38 -r /Solexa/ParsedData/PolIII/

**************************************************************************************
```

```
**************************************************************************************
**                          Eland Sequence Parser: June 2008                        **
**************************************************************************************
Parses sequence information from Eland Extended alignment summary files. For every
base, sums the quality scores generating a G, A, T, and C track xxx.bar file for 
visualization in IGB.  Also generates a consensus track (1-fraction consensus) for
each base.

-f The full path directory/file name of your xxx_export.txt(.zip) or
      xxx_sorted.txt(.zip) file(s).
-r Full path directory name for saving the results.
-g Full path directory name containing fasta files for reference base calling
      (e.g. chr1.fasta, chr5.fasta).
-v Versioned Genome (ie hg18, dm2, ce2, mm8), see UCSC Browser,
      http://genome.ucsc.edu/FAQ/FAQreleases.
-a Minimum aligment score, -10Log10(p-value), defaults to 13.
-c Minimum consensus score, -10Log10(p-value), defaults to 60.


Example: java -Xmx1500M -jar pathToUSeq/Apps/ElandSequenceParser -v hg18 -c 90
      -f /data/ExportFiles/ -r /data/Results -g /genomes/Hg18Fastas 

**************************************************************************************
```

```
**************************************************************************************
**                        Export Intergenic Regions    May 2007                     **
**************************************************************************************
EIR takes a gff file and uses it to mask a boolean array.  Parts of the boolean array
that are not masked are returned and represent integenic sequences. Be sure to put in
a gff line at the end of each chromosome noting the last base so you caputure the last
intergenic region. (eg chr1 GeneDB lastBase 3600000 3600001 . + . lastBase). Base
coordinates are assumed to be end inclusive, not interbase.

Parameters:
-g Full path file name for a gff file or directory containing such.
-t Base pairs to trim from the ends of each intergenic region, defaults to 0.
-m Minimum acceptable intergenic size, those smaller will be tossed, defaults to 60bp
-s Subtract one from the start and stop coordinates of your gff file.

Example: java -Xmx1000M -jar pathTo/T2/Apps/ExportIntergenicRegions -s -m 100 -g
                 /user/Jib/GffFiles/Pombe/sanger.gff

**************************************************************************************
```

```
**************************************************************************************
**                         Export Intronic Regions    June 2007                     **
**************************************************************************************
EIR takes a UCSC Gene table and fetches the most conservative/ smallest intronic
regions. Base coordinates are assumed to be end inclusive, not interbase.

Parameters:
-g Full path file name for the UCSC Gene table.
-m Minimum acceptable intron size, those smaller will be tossed, defaults to 60bp
-s Subtract one from the stop coordinates of your UCSC table to convert from interbase.

Example: java -Xmx1000M -jar pathTo/T2/Apps/ExportIntronicRegions -s -m 100 -g
                 /user/Jib/ucscPombe.txt

**************************************************************************************
```

```
**************************************************************************************
**                          FetchGenomicSequences: May 2008                         **
**************************************************************************************
Given a file containing genomic coordinates, fetches and saves the sequence (column
output: chrom origStart origStop fetchedStart fetchedStop completeFetch seq).

-f Full path file name to the file or directory containing tab delimited chrom, start,
        stop files.  Interbabase coordinates (zero based, end excluded).
-s Full path directory name containing containing genomic fasta files. The fasta
        defines the name of the sequence, not the file name. 
-b Fetch flanking bases, defaults to 0. Will set start to zero or end to last base if
        boundaries are exceeded.

Example: java -Xmx1000M -jar pathTo/T2/Apps/FetchGenomicSequences -f /data/miRNAs.txt
      -c /genomes/human/v35.1/ -b 5000. 


**************************************************************************************
```

```
**************************************************************************************
**                            Find Neighboring Genes:   Feb 2008                    **
**************************************************************************************
FNG takes a list of genes in UCSC Gene Table format and intersects them with a list of
regions finding the closest gene to each region as well as all of the genes that fall
within a given neighborhood. Distance is measured from the center of the region to the
transcription start site/ 1st base position in 1st exon. See Tables link under
http://genome.ucsc.edu/ . Note, output coordinates are zero based, end inclusive.

-g Full path file name for a tab delimited UCSC Gene Table (name chrom strand txStart
      txEnd cdsStart cdsEnd exonCount exonStarts exonEnds etc...) .
-p Full path file name for a tab delimited region list (chr, start, stop) .
-b Size of neighborhood in bp, default is 10000 
-c Only print closest genes.
-o Print neighbors on one line.

Example: java -jar pathTo/T2/Apps/FindNeighboringGenes -g /anno/hg17Ensembl.txt -p
      /affy/p53/finalPicks.txt -b 5000 -c

**************************************************************************************
```

```
**************************************************************************************
**                            File Cross Filter: March 2008                         **
**************************************************************************************
FCF take a column in the matcher file and uses it to parse the rows from other files.
Useful for pulling out and printing in order the rows that match the first file.

-m Full path file name for a tab delimited txt file to use in matching.
-f Ditto but for the file to parse, can specify a directory too.
-i Ignore duplicate keys.
-a Column index containing the unique matcher IDs, defaults to 0.
-b Ditto but for the files to parse.

Example: java -jar pathTo/T2/Apps/FileCrossFilter -f /extendedArrayData/ -m /old/
     originalArray.txt -a 2 -b 2

**************************************************************************************
```

```
**************************************************************************************
**                            File Match Joiner:  July 2008                         **
**************************************************************************************
FMJ loads a file and a particular column containing unique entries, a key, and then
appends the key line to lines in the parsed file that match a particular column.
Usefull for appending say chromosome coordinates to snp ids data, etc.

-k Full path file name for a tab delimited txt file (key) containing unique entries.
-f Ditto but for the file to parse, can specify a directory too.
-i Collapse duplicate keys.
-a Column index containing the unique key IDs, defaults to 0.
-b Ditto but for the files to parse.
-p Print only matches.

Example: java -jar pathTo/Apps/FileMatchJoiner -k /snpChromMap.txt -m /SNPData/
     --b 2 -p

**************************************************************************************
```

```
**************************************************************************************
**                             File Joiner: Feb 2005                                **
**************************************************************************************
Joins text files into a single file, avoiding line concatenations. This is a problem
with using 'cat * > combine.txt'.  Removes empty lines.

Required Parameters:
-f Full path name for the directory containing the text files.

Example: java -jar pathTo/T2/Apps/FileJoiner -f /affy/SplitFiles/

**************************************************************************************
```

```
**************************************************************************************
**                         File Splitter: June 2006                                 **
**************************************************************************************
Splits a big text file into smaller files given a maximum number of lines.

Required Parameters:
-f Full path file name for the text file.
-n Maximum number of lines to place in each split file.

Example: java -Xmx256M -jar pathTo/T2/FileSplitter -f /affy/bpmap.txt -n 50000

**************************************************************************************
```

```
**************************************************************************************
**                            Filter Point Data: Oct 2008                           **
**************************************************************************************
FPD drops or saves observations from PointData that intersect a list of regions
      (e.g. repeats, interrogated regions).

Options:
-p Point Data directories, full path, comma delimited. These should contain
      chromosome specific xxx.bar.zip files. 
-r Full path file name for a tab delimited text file containing regions to use in
      filtering the intersecting data (chr start stop ..., interbase coordinates).
-i Select data that intersects the list of regions, defaults to selecting data that
      doesn't intersect.
-a Acceptible intersection, fraction, defaults to 0.5
-s Shift centered position N bps 3' to accomodate chIP-seq fragment size. Stranded.
      Note, this is far less than 1/2 the expected fragment size, determine best
      value by visual inspection of likely positives. Defaults to 0. If you already
      shifted your data, be sure that filtering makes sense.

Example: java -Xmx1500M -jar pathTo/USeq/Apps/FilterPointData -p /data/PointData 
      -f /repeats/hg18RepeatMasker.bed -m 0.75 -s 73

**************************************************************************************
```

```
**************************************************************************************
**                                 Gr2Bar: Nov 2006                                 **
**************************************************************************************
Converts xxx.gr.zip files to chromosome specific bar files.

-f The full path directory/file name for your xxx.gr.zip file(s).
-v Genome version (ie hg18, dm2, ce2, mm8), get from UCSC Browser,
      http://genome.ucsc.edu/FAQ/FAQreleases

Example: java -Xmx1500M -jar pathTo/T2/Apps/Gr2Bar -f /affy/GrFiles/ -v hg17 

**************************************************************************************
```

```
**************************************************************************************
**                            Intersect Lists: May 2008                             **
**************************************************************************************
IL intersects two lists (of genes) and using randomization, calculates the
significance of the intersection and the fold enrichment over random. Note, duplicate
items are filtered from each list prior to analysis.

-a Full path file name for list A, one item per line.
-b Full path file name for list B, one item per line.
-t The total number of unique items from which A and B were drawn.
-n Number of permutations, defaults to 1000.
-p Print the intersection sets (common, unique to A, unique to B) to screen.

Example: java -Xmx1500M -jar pathTo/Apps/IntersectLists -a /Data/geneListA.txt -b 
     /Data/geneListB.txt -t 28356 -n 10000

**************************************************************************************
```

```
**************************************************************************************
**                         Intersect Regions: August 2008                           **
**************************************************************************************
IR intersects lists of regions (tab delimited: chrom start stop(inclusive)). Random
regions can also be used to calculate a p-value and fold enrichment.

-f First regions files, a single file, or a directory of files.
-s Second regions files, a single file, or a directory of files.
-g Max gap, defaults to 0. A max gap of 0 = regions must abut, negative values force
      overlap (ie -1= 1bp overlap, be careful not to exceed the length of the smaller
      region), positive values enable gaps (ie 1=1bp gap).
-e Score intersections where second regions are entirely contained by first regions.
-r Make random regions matched to the second regions file(s) and intersect with the
      first.  Enter the full path directory name containing chromosome specific
      interrogated regions files (ie named: chr1, chr2 ...: chrom start stop(inclusive)).
-c Match GC content of second regions file(s) when selecting random regions, rather
      slow. Provide a full path directory name containing chromosome specific genomic
      sequences.  To speed the matching place the fraction GC in the last column of
      your region file(s).
-n Number of random region trials, defaults to 1000.
-w Write intersection and difference files for the first and second region files.
-x Write paired intersections to file.
-p Print length distribution histogram for gaps between first and closest second.
-q Parameters for histogram, comma delimited list, no spaces:
       minimum length, maximum length, number of bins.  Defaults to -100, 2400, 100.
-i Serialized interval files are provided, not text region files.


Example: java -Xmx1500M -jar pathTo/Apps/IntersectRegions -f /data/miRNAs.txt
      -s /data/DroshaLists/ -g 500 -n 1000 -r /data/InterrogatedRegions/


**************************************************************************************
```

```
**************************************************************************************
**                      Make Splice Junction Fasta: August 2008                     **
**************************************************************************************
MSJF creates a multi fasta file containing sequences representing all possible linear
splice junctions. The header on each fasta is the chr_endPosExonA_startPosExonB. The
length of sequence collected from each junction is 2x the radius. A word of warning,
be very careful about the coordinate system used in the gene table to define the
start and stop of exons.  UCSC uses interbase and this is assumed in this app. Check
a few of the junctions to be sure correct splices were made. All junction sequences
are from the top/ plus strand of the genome, they are not reverse complemented.

Options:
-f Fasta file directory, should contain chromosome specific xxx.fasta files.
-u UCSC gene table, full path. See, http://genome.ucsc.edu/cgi-bin/hgTables
-s Sequence length radius.
-r Results fasta file, full path.

Example: java -Xmx1500M -jar pathTo/USeq/Apps/MakeSpliceJunctionFasta -s 32 
      -f /Genomes/Hg18/Fastas/ -u /Anno/Hg18/ucscKnownGenes.txt -r
      /Genomes/Hg18/Fastas/hg18_32_splices.fasta 

************************************************************************************
```

```
**************************************************************************************
**                         Make Transcriptome: October 2008                         **
**************************************************************************************
MT creates a multi fasta file containing sequences representing all possible linear
splice junctions and extended exons for each gene in the UCSC gene table. The size of
each junction is 2x the sequence length radius, the exon extension in 1x on each end.
Be very careful about the coordinate system used in the gene table to define the
start and stop of exons.  UCSC uses interbase and this is assumed in this app.
Sequences returned are the sense + strand. Note, ELAND does not like '.' in the fasta
headers so be sure these are not part of your gene names.

Options:
-f Fasta file directory, should contain chromosome specific xxx.fasta files.
-u UCSC gene table, full path. See, http://genome.ucsc.edu/cgi-bin/hgTables
-s Sequence length radius.
-r Results fasta file, full path.
-a Append gene coordinates onto name.

Example: java -Xmx1500M -jar pathTo/USeq/Apps/MakeTranscriptome -s 36 
      -f /Genomes/Hg18/Fastas/ -u /Anno/Hg18/ucscKnownGenes.txt -r
      /Genomes/Hg18/Fastas/hg18_32_splices.fasta 

************************************************************************************
```

```
**************************************************************************************
**                            Primer3 Wrapper: Dec  2006                            **
**************************************************************************************
Wrapper for the primer3 application. Extracts sequence, formats for primer3, executes
and parses the output to a spreadsheet. See http://frodo.wi.mit.edu/primer3/

-f Full path file name for your sequence file, tab delimited, sequence in 1st column.
-s Pick small product sizes (45-80bp), defaults to standard (80-150bp)
-p Full path name for the primer3_core application. Defaults to
     /nfs/transcriptome/software/noarch/T2/64Bit_Primer3_1.0.0/src/primer3_core
-m Full path file name for the mispriming library. Defaults to
     /nfs/transcriptome/software/noarch/T2/64Bit_Primer3_1.0.0/
     cat_humrep_and_simple.cgi.txt

Example: java -jar pathTo/T2/Apps/Primer3Wrapper -f /home/dnix/seqForQPCR.txt -p
    /nfs/transcriptome/software/noarch/T2/64Bit_Primer3_1.0.0/src/primer3_core
    -m /nfs/transcriptome/software/noarch/T2/64Bit_Primer3_1.0.0/
    cat_humrep_and_simple.cgi.txt -s 
**************************************************************************************
```

```
**************************************************************************************
**                           Print Select Columns: July 2006                        **
**************************************************************************************
Spread sheet manipulation.

Required Parameters:
-f Full path file or directory name for tab delimited text file(s)
-i Column indexs to print, comma delimited, no spaces
-n Number of initial lines to skip
-l Print only this last number of lines
-c Column word to append onto the start of each line
-r Append a row number column as the first column in the output file
-d Append file name onto the start of each line
-s Skip blank lines and those with less than the indicated number of columns.

Example: java -jar pathTo/T2/PrintSelectColumns -f /TabFiles/ -i 0,3,9 -n 1 -c chr

**************************************************************************************
```

```
**************************************************************************************
**                          Ranked Set Analysis: Jan 2006                           **
**************************************************************************************
RSA performs set analysis (intersection, union, difference) on lists of
genomic regions (tab delimited: chrom, start, stop, score, (optional notes)).

-a Full path file name for the first list of genomic regions.
-b Full path file name for the second list of genomic regions.
-d (Optional) Full path directory containing region files for all pair analysis.
-m Max gap, bps, set negative to force an overlap, defaults to -100
-s Save comparison as a PNG, default is no.

Example: java -jar pathTo/T2/Apps/RankedSetAnalysis -a /affy/nonAmpA.txt -b
      /affy/nonAmpB.txt -s

**************************************************************************************
```

```
**************************************************************************************
**                                ScanSeqs: Aug 2008                                **
**************************************************************************************
Takes chromosome specific PointData xxx.bar.zip files and uses a sliding window to
calculate smoothed window scores. These are saved as point and heat map/ stairstep
xxx.bar.zip graph files for direct viewing in the Integrated Genome Browser. If no
control data is provided, Bonferroni corrected p-values are estimated using a global
Poisson distribution. Otherwise, binomial p-values and Storey q-value FDRs are
estimated based on the treatment vs control window scores. Lastly, empirical FDRs can
be estimated by generating a null distribution of control vs control enriched regions
and scoring each treatment - control threshold relative to the null.  In this case,
one should provide >2x the # of control reads relative to the treatment reads to
prevent significant sub sampling of the treatment observations.

Options:
-s Save directory, full path.
-t Treatment Point Data directories, full path, comma delimited. These should
       contain chromosome specific xxx.bar.zip files. If needed, strands will be
       merged. 
-c Control Point Data directories, ditto. Optional but highly recommended to avoid
       systematic bias.
-l Enter # of mappable bases, typically 0.9 * bp size genome for complex organisms.
       Human 3253037807 * 0.9 = 2927734026
       S.cerevisiae 12162996 * 0.99 = 12041366
       See 'All genomes' http://www.ensembl.org for genome sizes.
-r Full path to R with Storey's q-value library.
-w Window size in bps, defaults to 300.
-m Minimum number window reads, defaults to 10. Setting this less than 10 will
       compromise the qvalue estimation. 
-e Estimate empirical FDRs, provide 2x control data.
-f Filter windows with high read control read counts.
-g Maximum control window read count, defaults to 50.
-j Remove negative scoring windows when saving window data. Recommended for chIP-seq.
-a Don't save heat map graphs.
-b Don't save point graphs.
-d Don't save normalized difference graphs.

Example: java -Xmx1500M -jar pathTo/USeq/Apps/ScanSeqs -t
      /Data/PolIIRep1/,/Data/PolIIRep2/ -c /Data/Input1/,Data/Input2/ -s
      /Data/PolIIResults -w 250 -r /usr/bin/R -b -j -e

**************************************************************************************
```

```
**************************************************************************************
**                           Score Chromosomes: Sept 2008                           **
**************************************************************************************
SC scores chromosomes for the presence of transcription factor binding sites. Use the
following options:

-g The full path directory name to the split genomic sequences (i.e. chr2L.fasta, 
      chr3R.fasta...), FASTA format.
-t Full path file name for the FASTA file containing aligned trimmed examples of
      transcription factor binding sites.  A log likelihood position specific
      probability matrix will be generated from these sequences and used to scan the
      chromosomes for hits to the matrix.
-s Score cut off for the matrix. Defaults to the score of the lowest scoring sequence
      used in making the LLPSPM.
-p Print hits to screen, default is no.
-v Provide a versioned genome (ie hg18, dm2, ce2, mm8), see UCSC Browser,
      http://genome.ucsc.edu/FAQ/FAQreleases, if you would like to write graph LLPSPM
      scores to bar files for direct viewing in IGB.

Example: java -Xmx4000M -jar pathTo/T2/Apps/ScoreChromosomes -g /my/affy/Hg18Seqs/ -t 
      /my/affy/fgf8.fasta -s 4.9 -v H_sapiens_Mar_2006

**************************************************************************************
```

```
**************************************************************************************
**                           ScoreParsedBars: Sept 2008                             **
**************************************************************************************
For each region finds the underlying scores from the chromosome specific bar files.
Prints the scores as well as their mean . A p-value for each region's score can be
calculated using chromosome, interrogated region, length, # scores, and gc matched
random regions. Be sure to set the -u flag if your scores are log2 values.

-r Full path file name for your region file (tab delimited: chr start stop(inclusive)).
-b Full path directory name for the chromosome specific data xxx.bar files.
-o Bp offset to add to the position bar file coordinates, defaults to 0.
-s Bp offset to add to the end of each region, defaults to 0.
-u Unlog the bar file values, set this flag if your scores are log2 transformed.
-g Estimate a p-value for the score associated with each region. Provide a full path
         directory name for chromosome specific gc content boolean arrays. See
         ConvertFasta2GCBoolean app. Complete option -i
-i If estimating p-values, provide a full path file name containing the interrogated
         regions (tab delimited: chr start stop ...) to use in drawing random regions.
-n Number of random region sets, defaults to 1000.
-d Don't print individual scores to screen.

Example: java -jar pathTo/Apps/ScoreParsedBars -b /BarFiles/Oligos/
       -r /Res/miRNARegions.bed -o -30 -s -60 -i /Res/interrRegions.bed
       -g /Genomes/Hg18/GCBooleans/

**************************************************************************************
```

```
**************************************************************************************
**                           Score Sequences: July 2007                             **
**************************************************************************************
SS scores sequences for the presence of transcription factor binding sites. Use the
following options:

-g The full path FASTA formatted file name for the sequence(s) to scan.
-t Full path file name for the FASTA file containing aligned trimmed examples of
      transcription factor binding sites.  A log likelihood position specific
      probability matrix will be generated from these sequences and used to scan the
      sequences for hits to the matrix.
-s Score cut off for the matrix. Defaults to zero.

Example: java -Xmx500M -jar pathTo/T2/Apps/ScoreSequences -g /my/affy/DmelSeqs.fasta
      -t /my/affy/zeste.fasta

**************************************************************************************
```

```
**************************************************************************************
**                               Sgr2Bar: July 2008                                 **
**************************************************************************************
Converts xxx.sgr(.zip) files to chromosome specific bar files.

-f The full path directory/file name for your xxx.sgr(.zip) file(s).
-v Genome version (ie H_sapiens_Mar_2006, M_musculus_Jul_2007), get from UCSC Browser.
-s Strand, defaults to '.', use '+', or '-'
-t Graph file should be viewed as a stair step, defaults to bar

Example: java -Xmx1500M -jar pathTo/Apps/Sgr2Bar -f /affy/sgrFiles/ -s + -t
      -v D_rerio_Jul_2006

**************************************************************************************
```

```
**************************************************************************************
**                            SubSamplePointData: April 2008                        **
**************************************************************************************
SSPD takes PointData directories and randomly selects points from each directory and
saves the merge.

-f Comma delimited full path PointDataDirectories from which to draw or a single 
       directory containing multiple PointDataDirectories.
-n Total number of observations desired.
-s Full path file directory in which to save the results.
-p Take an equal percent of reads from each PointData directory instead of the same #.
-m Minimum number observations in each directory to include in sub sampling.

Example: java -Xmx1500M -jar pathTo/USeq/Apps/SubSamplePointData -n 10000000 -p -f
    /Data/WCE1_Point,/Data/WCE2_Point,/Data/WCE3_Point -s /Data/Sub/ 

**************************************************************************************
```

```
**************************************************************************************
**                               Tag2Point: August 2008                             **
**************************************************************************************
Splits and converts tab delimited text (chr start stop ... strand (+ or -)) text
files into center position binary xxx.bar files. Use the appropriate options
to convert your coordinates into interbase coordiantes (zero based, end excluded).

-v Versioned Genome (ie hg18, dm2, ce2, mm8), see UCSC Browser,
      http://genome.ucsc.edu/FAQ/FAQreleases.
-i Strand column index, defaults to 5. 1st column is zero.
-b Subtract one from the beginning of each region.
-e Add one to the end of each region.
-s Shift centered position x bps 3' to accomodate chIP-seq fragment size. Stranded.
      Note, this is far less than 1/2 the expected fragment size, determine best
      value by visual inspection of clear positives.
-f The full path directory/file name of your text file(s).

Example: java -Xmx1500M -jar pathTo/T2/Apps/Tag2Point -f /Solexa/BedFiles/
     -v hg18 -b -s 38

**************************************************************************************
```

```
**************************************************************************************
**                                 QCSeqs: Jan 2008                                 **
**************************************************************************************
QCSeqs takes directories of chromosome specific PointData xxx.bar.zip files that 
represent replicas of signature sequencing data, merges the strands, uses a sliding
window to sum the hits, and calculate Pearson correlation coefficients for the window
sums between each pair of replicas.  Only windows with a sum score >= the minimum 
are included in the correlation.

-d Split chromosome Point Data directories, full path, comma delimited. (These should
       contain chromosome specific xxx.bar.zip files). 
-t Temp directory, full path. This will be created and then deleted.
-w Window size in bps, defaults to 500.
-s Window step size in bps, defaults to 250.
-m Minimum window sum score, defaults to 5.

Example: java -Xmx1500M -jar QCSeqs -d /Solexa/PolII/Rep1PntData/,/Solexa/PolII/
      /Rep2PntData/ -t /Solexa/PolII/TempDelMe -w 250 -s 50 

**************************************************************************************
```

```
**************************************************************************************
**                               Wig2Bar: July 2008                                 **
**************************************************************************************
Converts variable step and fixed step xxx.wig(Var) files to chrom specific bar files.

-f The full path directory/file name for your xxx.wig(Var) file(s).
-v Genome version (ie hg18, dm2, ce2, mm8), get from UCSC Browser,
      http://genome.ucsc.edu/FAQ/FAQreleases
-s Skip wig lines with designated value/score.

Example: java -Xmx1500M -jar pathTo/Apps/Wig2Bar -f /WigFiles/ -v hg18 -s 0.0 

**************************************************************************************
```
