# Supplementary material for: Empirical methods for controlling false positives and estimating confidence in ChIP-Seq peaks
Source: BMC Bioinformatics. 2008 Dec 5;9:523. doi: 10.1186/1471-2105-9-523 (PMC2628906; doi:10.1186/1471-2105-9-523)
Supplement: Additional File 1 — A variety of html documents from the USeq web site detailing the available applications, their best usage, output file type descriptions, command line menus, etc. [file 1471-2105-9-523-S1.zip › USeqUserGuides/outputFileTypeDescriptions.html]

USeq Output File Type Descriptions


# USeq results typically include:

- Two data folders containing information relevant to **Windows** and **Enriched Regions**:

  - **PointData** These folders contain mapped read data split by chromosome and strand. For each read, its center position, and any associated score is saved. These are used by various USeq applications to perform analyisis. They can be viewed in IGB but be aware that PointData that share the same position are graphed on top of each other in IGB, not summed.

    - **Windows:** several different measurements related to the overlapping window scanning summary statistics. These are best used for visualization in the IGB browser. For each summary score, two types of window representations are given.
      **HeatMap** is a window representation best viewed in IGB as a stair step or black and white heatmap.
      **Point** window summaries are where the window score is assigned to the center position in the window. Both use the xxx.bar.zip binary format.

      - **NormDiff**: A normalized difference window score = (Sum T - Sum C)/ ((Sum T + Sum C) ^ 0.5)- **PVal**: -10Log10(p-values) - Bionomial p-values for each window calculated by comparing the number of treatment reads to the number of control reads. Not multiple test corrected.- **QValFDR**: -10Log10(q-values) - Window level bionomial p-values converted into q-value FDRs using John Storey's R package.- **EmpFDR**: -10Log10(FDR) - Empirical false discovery rates based on a control vs control null distribution.- **Total**: A sum of the window point data, only provided when a treatment only analysis is run.- **BCorPVal**: -10Log10(p-values), Bonferoni corrected global Poisson p-values for each window, only provided when a treatment only analysis is run. Not reliable.- **xxx.swi**: Serialize window object array used by the EnrichedRegionMaker application.

      - **Enriched Regions:** Enriched Regions are collapsed overlapping windows best used for downstream analysis. Several different sets of ERs can be created by specifying multiple thresholds or asking the application to produce the top 100, 200, 400, etc. ERs.
        For each ER set, a folder (name\_threshold\_#ERs) is created containing the following.

        - **xxx.xls**: a spreadsheet report for the ER set, lots of info, these are ordered by the the best window score with the most significant on top.- **xxx.egr**: a multiple score EGR format file for viewing in IGB.- **xxx.gff**: a GFF file- **XXXbpSubWinData**: If treatment and control data were provided to the EnrichedRegionMaker, each ER is rescanned using a small window to identify the best peaks within the ER. These are included in the spreadsheet report as well as outputed as graph bar files, xxx.egr, and xxx.gff files.
