# Supplementary material for: Empirical methods for controlling false positives and estimating confidence in ChIP-Seq peaks
Source: BMC Bioinformatics. 2008 Dec 5;9:523. doi: 10.1186/1471-2105-9-523 (PMC2628906; doi:10.1186/1471-2105-9-523)
Supplement: Additional File 1 — A variety of html documents from the USeq web site detailing the available applications, their best usage, output file type descriptions, command line menus, etc. [file 1471-2105-9-523-S1.zip › USeqUserGuides/README.html]

README


# USeq

## Disclaimer:

This software is distributed with no warrantee or guarantee of fitness. See the open source BSD License

## What is USeq?

USeq is a collection of software tools for for both low and high level analysis of next generation, ultra high throughput signature sequencing data from the Solexa, SOLiD, and 454 platforms.
Initial emphasis: chIP-seq with FDR estimation, QC, set analysis, neighboring genes, and remapping data. USeq is under continuous development at the 
Huntsman Cancer Institute.

## How to use USeq?

To use USeq, download the latest release, install Java 1.5+, R and the Q-Value library (see below) and launch the java jar applications with the -jar option.
For example, to execute 'ScanSeqs' type 'java -jar yourPathTo/USeq/Apps/ScanSeqs' on the command line to print a menu and description of options.

## Requirements

- Java 1.5+- R with the Q-Value library- Familiarity with command line programing

## Documentation:

- Usage - An outline/ tutorial on how to use USeq to process your sequencing data.
- Applications - A listing of the various USeq applications.
- Menus - Command line menus/ instructions/ parameters for each of the applications.
- Output File Type Descriptions - Description of the different types of file output generated by USeq applications.
- JavaDocs - Code documentation for BioTools which includes USeq. Eclipse users can directly import the BioTools directory as an Eclipse project.
- BSD License - The BSD open source USeq license.

## Common mistakes:

- No java sourced or installed. Type 'java -version' on the command line. It should be 1.5+
- Not using the -jar option. Type: 'java -jar yourPathTo/USeq/Apps/ScanSeqs'
- Out of memory error. Increase the heap size with the -Xmx flag. Type: 'java -jar -Xmx1500M yourPathTo/USeq/Apps/ScanSeqs'
- Trying to run the apps on Windows. It is worth a try. These have been tested on Linux and Mac OSX.

Don't hesitate to contact David Nix in the Bioinformatics Core with questions or comments.

Many thanks to Ken Boucher in the Biostatistics Shared Resource for help with the statistical methods.

Want to contribute? Join the open source project at USeq: SourceForge
