# Supplementary material for: Empirical methods for controlling false positives and estimating confidence in ChIP-Seq peaks
Source: BMC Bioinformatics. 2008 Dec 5;9:523. doi: 10.1186/1471-2105-9-523 (PMC2628906; doi:10.1186/1471-2105-9-523)
Supplement: Additional File 1 — A variety of html documents from the USeq web site detailing the available applications, their best usage, output file type descriptions, command line menus, etc. [file 1471-2105-9-523-S1.zip › USeqUserGuides/usage.html]

Usage

# USeq Usage

  

This is an outline on how to use USeq to process your ChIP-seq data. Other protocols to follow.

The applications are designed to be user friendly and require only moderate familiarity
with command line programs. When in doubt type 'java -jar pathTo/USeq/Apps/ApplicationName' to pull a menu of options.
See the readme file for installation instructions.

## ChIP Recommendations

1. Use a robust chIP protocol/ kit such as those from  Active Motif, Epigentek, CellSignaling, USB, Imgenex, ...- If known positives and negatives are available, perform qPCR to demonstrate enrichment for these regions and/ or optimize your chIP protocol.- If not available, consider making use of several of the commercially available control kits/ antibodies.- For each antibody:
         1. Perform 4+ independent sample preparations from the same source material (e.g. wash and crosslink 30 10cm tc plates containing a cell line, lyse, pool all plates, split in 4 parts, and proceed with 4 independent sample preps). The idea here is that regions chIPed in all samples will be preferentially weighted, those pulled from one prep, down weighted.- When possible, fragment your sample using enzymatic digestion. Sonication, although commonly used, is biased and requires considerable optimization. The target size range is 200-500bp. Confirm and document it on a gel.- Do not size select the IP material, this will be performed after sequencing linker ligation.- Pool the IPed material and clean up with a column especially if phenol was used in the preparation.- Quantify and size the clean fragmented IPed DNA using a PicoGreen assay.- A minimum of 5-10ng is required for sequencing. Much more (50-100ng) should be prepared and saved for qPCR validation. If you have less than 5ng of DNA, consider adding input DNA to make up the difference, otherwise a PCR bottleneck effect will likely introduce numerous false positives during the library preparation. Alternatively, don't pool the biological replicas and run each individually.- Submit the prepped chIP DNA as well as 50ng of Input DNA that has been processed in the same way as the chIP DNA sans the IP (e.g. fragmented, cleaned, quantified). Sequenced input data is likely reusable provided the sample prep protocol has not changed.- If you are using multiple antibodies to chIP various targets from the same source, only submit one pooled input sample. This will be used to control for systematic bias for all of your chIP samples.- For higher eukaryotes, 10 million mapped reads per sample are recommended for a whole genome analysis. This equates to ~4 lanes on the Solexa GAI.- We strongly recommend running just one lane for each newly prepped sample to see if the prep worked and whether high quality data can be generated. After the sample has passed all QC metrics, then run subsequent lanes with the old sample. Unlike with microarray experiments, runs from different days/months are just as good and can be added to prior data.

## Required Computer Resources:

1. Java 1.5+- R with the Q-Value library- These command line applications have been tested on MacOSX and Linux. They have not been tested on a Windows machine. All the source code, with extensive documentation, is included in the USeq package.- A 64 bit computer with > 4-8 GigaBytes RAM.

## Basic ChIP-Seq Low Level Data Analysis

1. Align your sequences to a reference genome using your aligner of choice.- Convert your alignments into binary PointData using the ElandParser or the Tag2Point application.- Consider removing reads that intersect known false positive regions (e.g. satellite repeats) with the FilterPointData application.- For comparison purposes, you may want to sub sample particular PointData to match the number of reads in other datasets using the SubSamplePointData application. Note, this is automatically performed in ScanSeqs.- If you have replica data, compare the replicas using QCSeqs to calculate a correlation coefficient.- Run ScanSeqs to window scan your data. Choices here include:
             - Window size - typically set this to the size of your fragmented chIP sample.- Minimum number window reads - There is a trade off here. This needs to be set to be set to >= 10 to enable correct conversion of p-values using Storey's q-value FDR method. Unfortunately, this may exclude too many regions from low density dataset analysis (e.g. higher eukaryote datasets with < 10 million treatment+control mapped reads). Some options, set to 1 and ignore the QValFDR values. Increase the size of the window. Sequence more lanes.- Estimate empirical FDRs - Again another trade off. This method is more accurate and robust compared to the q-value method but requires 2x the number of input control reads as the number of chIP reads. If you select this option and don't have 2x the input reads, ScanSeqs with subsample your chIP reads to make this ratio.- Filter windows with high control read counts - Useful for reducing false positives in chIP-seq analysis. Error on the high side here.- Various options to not save different graph types - Writing out these bar files is the slowest part of ScanSeqs. Only write out what you need. Eliminate entirely when generating preliminary data.- Merge overlaping windows into binding peaks and print several reports using the EnrichedRegionMaker. If you used the empirical FDR estimation option in ScanSeqs, a good starting threshold is 13 a -10Log10(FDR = 5%). This is rather accurate and slightly conservative.- Use the DefinedRegionScanSeqs to perform a gene centric or your favorite regions analysis.

## ChIP-Seq High Level Data Analysis Options

1. Find the neariest neighboring genes to a list of binding peaks using FindNeighboringGenes- Intersect your list of binding peaks to another with IntersectRegions- Perform a directed ScanSeqs using a gene table or list of regions, see DefinedRegionScanSeqs- Fetch the sequence under each of your binding peaks using FetchGenomicSequences- Score sequences and chromosomes for binding sites with ScoreSequences and ScoreChromosomes- Make primers for qPCR validation using the Primer3Wrapper

Be sure to check out the full listing of applications. They might save you days of coding.

Questions? Comments? Contact David Nix in the Bioinformatics Core.

Many thanks to Ken Boucher in the Biostatistics Shared Resource for help with the statistical methods.
